# UPLC‐MS/MS‐based plasma lipidomics reveal a distinctive signature in systemic lupus erythematosus patients

**DOI:** 10.1002/mco2.67

**Published:** 2021-04-29

**Authors:** Jiaxi Chen, Chong Liu, Shenyi Ye, Ruyue Lu, Hongguo Zhu, Jiaqin Xu

**Affiliations:** ^1^ Taizhou Hospital of Zhejiang Province Affiliated to Wenzhou Medical University Taizhou China

**Keywords:** biomarkers, lipidomics, systemic lupus erythematosus

## Abstract

Global lipidomics is of considerable utility for exploring altered lipid profiles and unique diagnostic biomarkers in diseases. We aim to apply ultra‐performance liquid chromatography‐tandem mass spectrometry to characterize the lipidomics profile in systemic lupus erythematosus (SLE) patients and explore the underlying pathogenic pathways using the lipidomics approach. Plasma samples from 18 SLE patients, 20 rheumatoid arthritis (RA) patients, and 20 healthy controls (HC) were collected. A total of 467 lipids molecular features were annotated from each sample. Orthogonal partial least square‐discriminant analysis, K‐mean clustering analysis and Kyoto Encyclopedia of Genes and Genomes pathway analysis indicated disrupted lipid metabolism in SLE patients, especially in phospholipid, glycerol, and sphingolipid metabolism. The area under curve (AUC) of lipid metabolism biomarkers was better than SLE inflammation markers that ordinarily used in the clinic. Proposed model of monoglyceride (MG) (16:0), MG (18:0), phosphatidylethanolamine (PE) (18:3–16:0), PE (16:0–20:4), and phosphatidylcholine (PC) (O‐16:2–18:3) yielded AUC 1.000 (95% CI, 1.000–1.000), specificity 100% and sensitivity 100% in the diagnosis of SLE from HC. A panel of three lipids molecular PC (18:3‐18:1), PE (20:3–18:0), PE (16:0–20:4) permitted to accurately diagnosis of SLE from RA, with AUC 0.921 (95% CI, 0.828–1.000), 70% specificity, and 100% sensitivity. The plasma lipidomics signatures could serve as an efficient and accurate tool for early diagnosis and provide unprecedented insight into the pathogenesis of SLE.

## Graphical abstract

Lipidomics profiling workflow. (a) Plasma lipidomics extraction was accomplished by fractionating samples after addition internal standard. (b) UPLC‐MS/MS‐based platforms were used to perform optimal profiling of plasma lipidomics. Normalization was carried out with quality control (QC) calibration and internal standard correction. (c) Univariate and multivariate data analyses were performed. (d) Selection of the best candidate biomarkers for the early diagnosis of SLE.

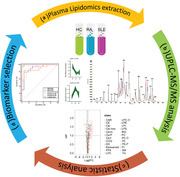

## 1 INTRODUCTION

Systemic lupus erythematosus (SLE) is a complex autoimmune inflammation disease, associated with complement dysregulation, interferon activation, and immune complexes deposited in multiple organs such as skin, joints, blood vessels, and kidney.^1^, ^2^ SLE develops with an incidence of 7.4–159.4 cases per 100,000 individuals worldwide and affects women at childbearing age predominantly.^3^, ^4^ Unfortunately, SLE is difficult to diagnose, check or treat, and there is no specific cure for this disease, only drugs that relieve symptoms and slow down the disease progression.^5^ Anti‐double stranded DNA antibody showed high specificity (92%–96%), but sensitivity is very moderate for SLE (only 57%–67%).^6^ Therefore, identification of SLE early diagnostic biomarkers and making it a useful new tool are unmet medical needs.

Lipid molecules play an important role in the energy storage and the formation of membrane structures and serve as secondary messengers in signaling that affect cell proliferation, signal transduction, and apoptosis.^7^, ^8^ Previous lipidomic studies have shown the potential of plasma lipidomic profiling in the diagnosis of cancers,^9^ type 2 diabetes,^10^ cardiovascular disease,^11^ and so on. However, few reports exist at present regarding the quantification of lipid molecules and large‐scale profiling and involve comprehensively studying lipid pathways and statistical analysis in SLE.

Due to the enhanced separation efficiency, ultra‐performance liquid chromatography‐tandem mass spectrometry (UPLC‐MS/MS) was considered as the appropriate technique for large‐scale lipidomics study. As the common pathogenesis and genome‐wide association studies (GWASs) revealed some common single nucleotide polymorphisms related to both rheumatoid arthritis (RA) and SLE.^12^, ^13^ We compared SLE patients with RA patients and healthy controls (HCs) in our study to explore whether plasma lipidomics will provide valuable information regarding the potential diagnostic biomarkers as well as the physiology in SLE.

## 2 RESULTS

### 2.1 Characteristics of participants

The clinical features and demographic of the study cohort are summarized in Table 1. Comparisons data of gender, age, and BMI were not significantly different between the three groups (*p *> 0.05). Comparisons data of the biochemical and immunity indicated higher levels of antistreptolysin O, and dsDNA in SLE patients.

**TABLE 1:** Demographic and clinical characteristics of the validation cohort

Variable	Control (*n* = 20)	RA (*n* = 20)	SLE (*n* = 18)
Age, mean ± SD (years)	40.2 ± 2.9	45.7 ± 3.2	39.1± 3.3
Males, *n* (%)	5 (25)	5 (25)	3 (17)
BMI, mean ± SD (Kg/m^2^)	20.9 ± 0.5	22.9 ± 0.7	22.1 ±0.5
**SLEDAI, *n* (%)**			
Inactive (0–11)	–	–	10 (55)
Active (>11)	–	–	8 (45)
**Biochemistry, mean ± SD**			
IGG (g/L)	17.8 ±4.8	13.5 ± 0.5	12.2 ± 1.1
IGA (g/L)	2.3 ± 0.2	3.2 ± 0.4	2.2 ± 0.4^b^
IGM (g/L)	1.3 ± 0.1	1.1 ± 0.1	1.0 ±0.1^a^
C3 (g/L)	1.2 ± 0.5	1.3 ± 0.6	1.0 ± 0.6^b^
C4 (g/L)	0.3 ± 0.2	0.3 ± 0.1	0.3 ± 0.1
C1q (mg/L)	210.4 ± 8.4	221.6 ± 9.6	179.3 ± 8.3^ab^
CRP (mg/L)	2.6 ± 1.4	16.4 ± 8.0^a^	15.3 ± 8.7^b^
ASO (KIU/L)	18.8 ± 26.7	45.2 ± 12.3^a^	30.2 ± 5.9^a^
RF (IU/L)	12.4 ±2.7	131.3 ±29.2^a^	7.2 ± 0.7^b^
dsDNA	21.5 ± 6.0	45.9 ±15.8^a^	128.6 ± 39.0^ab^

Abbreviations: ASO, antistreptolysin O; BMI, body mass index; C1q, complement Clq; C3, complement 3; C4, complement; CRP, C‐reactive protein; dsDNA, anti‐double stranded DNA; IGA, immunoglobulin A; IGG, immunoglobulin G; IGM, immunoglobulin M; RF, rheumatoid factors；

“‐”, not avialible; ^a^, *p *< 0.05 compared with control; ^b^, *p *< 0.05 compared with RA, by the Bonferroni method of multiple‐range testing.

### 2.2 Comparative plasma lipid metabolomic profiles and pathway analysis between SLE patients and HCs

**FIGURE 1:**
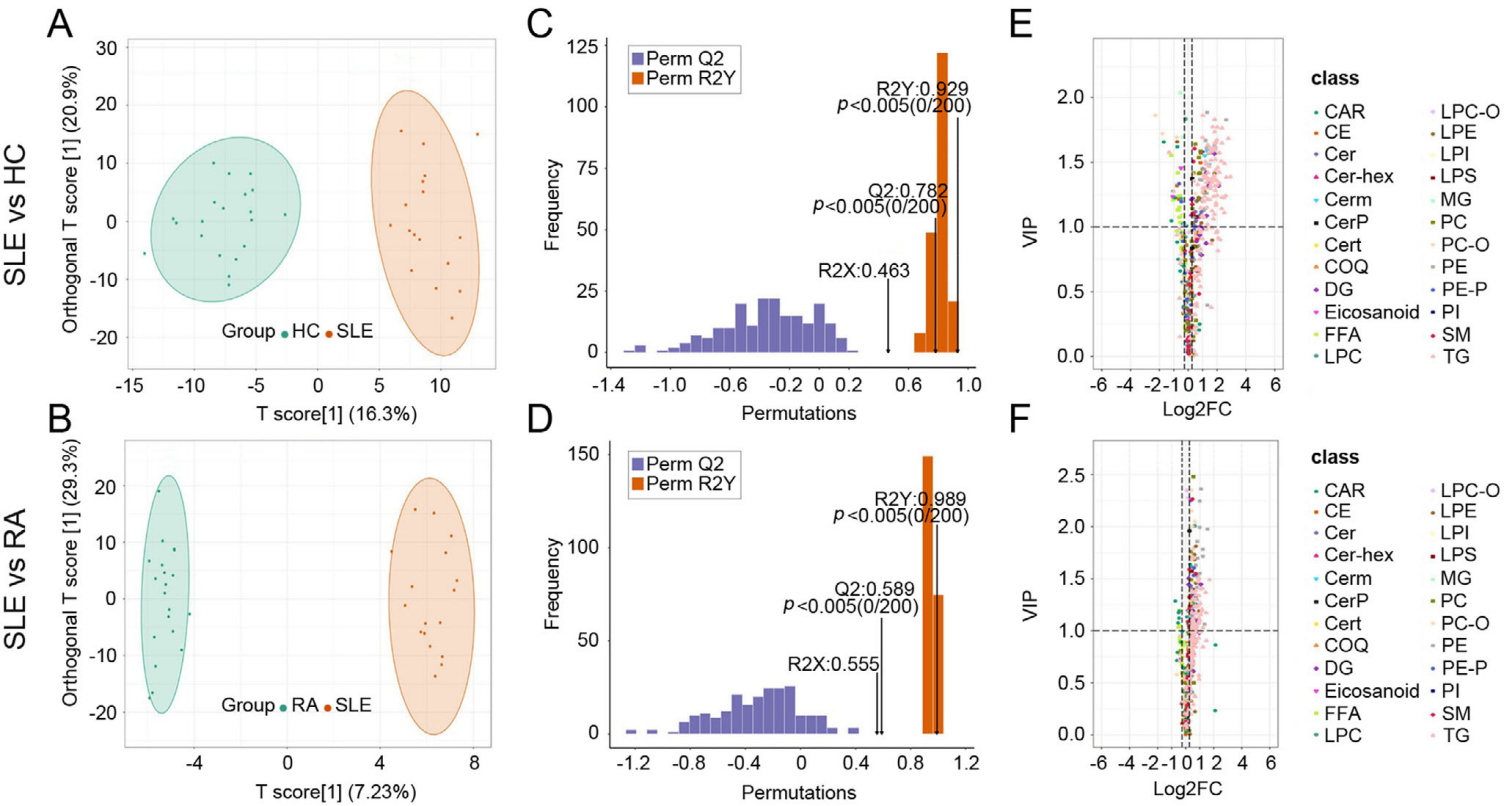
Establishment and validation of OPLS‐DA models (SLE group vs. HC group, SLE group vs. RA group) and screening of differential lipids. (A and B) The OPLS‐DA models showed that the lipid metabolites between SLE group and HC group, SLE group and RA group were separated clearly. (C and D) The models had good prediction ability between SLE group and HC group, SLE group and RA group. (E and F) The difference lipid category between SLE group and HC group, SLE group and RA group were identified by volcanic maps

A total of 467 lipids in plasma were identified in both patients and healthy cohorts. The supervised orthogonal partial least square‐discriminant analysis (OPLS‐DA) model revealed a clear segmentation between SLE patients and HCs (Figure 1A). The verification results of the permutation test to the OPLS‐DA model showed that R2X was 0.463, R2Y was 0.929, and Q2 was 0.782 respectively (*p* < 0.005) (Figure 1C). Differential lipids were screened by using a fold change > 1.2 or < 1/1.2, and variable importance projection (VIP) ≥ 1.0, were showed in the volcano plot (Figure 1E). A total of 191 lipids had significantly changed in SLE patients versus HCs, of which 157 upregulated and 34 downregulated lipids. The area under curve (AUC) of 22 lipids was higher than 0.85. Among them, 19 lipids were upregulated, and four lipids were downregulated. Monoglyceride (MG) (16:0) decreased significantly in SLE patients, with the AUC area 0.997, specificity 95% and sensitivity 100%, respectively (Table 2).

**TABLE 2:** Diagnostic capacity of the 22 selected lipid metabolites altered in SLE compared to HC

Metabolite		SEN (%)	SPE (%)	AUC	*p* value	Type
MG	MG(16:0)	100	95	0.997	0.000	down
	MG(18:0)	94	80	0.906	0.000	down
PE	PE(18:3‐16:0)	94	85	0.944	0.000	up
	PE(16:0‐20:4)	71	100	0.926	0.000	up
	PE(16:0‐18:0)	95	75	0.882	0.000	up
	PE(16:1‐18:0)	76	90	0.879	0.002	up
	PE(18:1‐16:0)	71	90	0.879	0.006	up
	PE(20:3‐18:0)	71	90	0.873	0.007	up
TG	TG(52:0) NL‐16:0	100	80	0.953	0.000	up
	TG(50:0) NL‐18:0	82	95	0.944	0.000	up
	TG(50:0) NL‐16:0	100	80	0.938	0.000	up
	TG(50:3) NL‐18:3	82	90	0.921	0.001	up
	TG(52:0) NL‐14:0	100	70	0.907	0.000	up
	TG(50:1) NL‐16:0	76	95	0.906	0.000	up
	TG(48:0) NL‐16:0	71	90	0.901	0.001	up
	TG(52:1) NL‐18:1	82	85	0.888	0.001	up
	TG(56:5) NL‐16:0	88	75	0.882	0.000	up
	TG(56:5) NL‐22:4	96	60	0.881	0.000	up
	TG(52:1) NL‐20:1	65	95	0.879	0.001	up
	TG(52:3) NL‐18:0	91	90	0.873	0.000	up
LPE	LPE(24:6)	82	85	0.903	0.000	down
PC	PC(O‐16:2‐18:3)	95	85	0.897	0.001	down

Abbreviations: AUC, area under curve; MG, monoglyceride; LPE, lysophosphatidylethanolamine; PC, phosphatidylcholine; PE, phosphatidylethanolamine; SEN, sensitivity; SPE, specificity; TG: triglycerides.

“:”, carbons and double bonds; “‐”, fatty acyle constituents.

**FIGURE 2:**
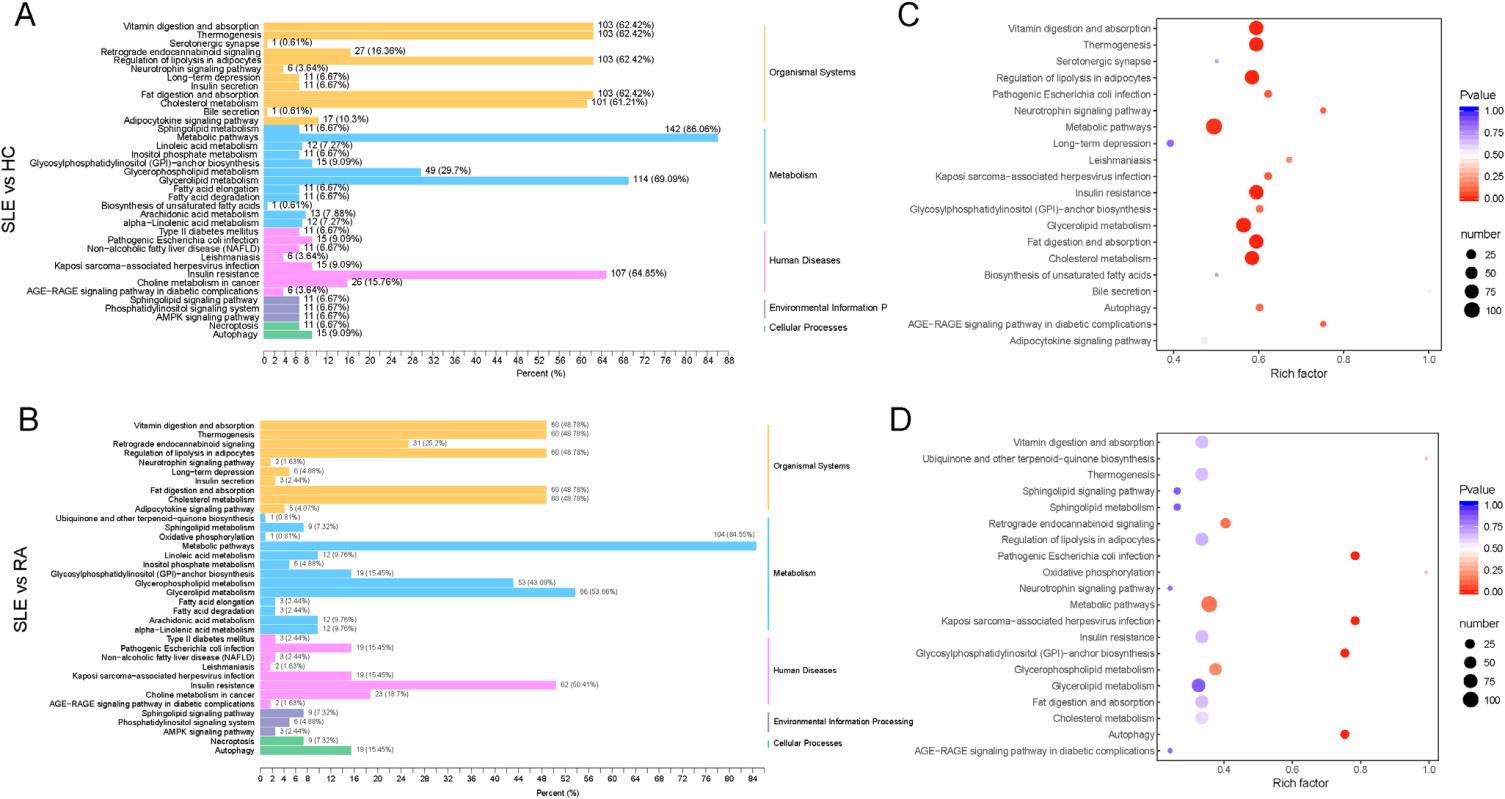
The lipidome KEGG enrichment analysis of SLE patients. (A and C) KEGG pathway analysis of differentially expressed lipids between SLE and HC groups. (B and D) KEGG pathway analysis of differentially expressed lipids between SLE and RA groups. The color of bubbles represents the value of adjusted *p*‐value, and the size of bubbles represents the number of counts

Kyoto Encyclopedia of Genes and Genomes (KEGG) pathway analysis was used to check the lipid metabolomic pathways that were affected in SLE patients. The differential metabolites are enriched in the following pathways, such as vitamin digestion absorption, thermogenesis, regulation of lipolysis in adipocytes, metabolic pathways, insulin resistance, glycerolipid metabolism, fat digestion and absorption, and cholesterol metabolism (Figures 2A and 2B).

### 2.3 Comparative plasma lipid metabolomic profiles and pathway analysis between SLE and RA patients

The supervised OPLS‐DA model revealed a clear segmentation between RA and SLE patients (Figure 1B). Specifically, OPLS‐DA verification chart revealed the prediction ability of the model (Q2), goodness of fit (R2X and R2Y) were 0.589, 0.555 and 0.989, respectively (Figure 1D). Differential lipids were screened by using a fold change > 1.2 or < 1/1.2, and VIP ≥ 1.0 and showed in the volcano plot (Figure 1F). Based on this standard, a total of 164 differentially lipid metabolomics were identified between RA and SLE patients (including 156 upregulated and eight downregulated lipids). The AUC scores of 14 lipids panel were significantly higher than 0.8. Among them, phosphatidylcholine (PC) (18:3–18:1) could be used for accurately diagnosis of RA from SLE, with 0.897 AUC, 80% specificity and 95% sensitivity (Table 3).

**TABLE 3:** Diagnostic capacity of the 14 selected lipid metabolites altered in SLE compared to RA

Metabolite		SEN (%)	SPE (%)	AUC	*p* value	Type
PC	PC(18:3‐18:1)	95	80	0.897	0.000	up
	PC(O‐12:0‐14:0)	95	65	0.859	0.000	up
	PC(O‐18:3‐20:2)	100	60	0.851	0.000	up
	PC(O‐18:2‐18:2)	100	60	0.821	0.001	up
	PC(O‐20:3‐20:2)	95	60	0.818	0.001	up
	PC(O‐18:3‐18:2)	100	50	0.803	0.000	up
PE	PE(20:3‐18:0)	88	85	0.876	0.000	up
	PE(16:0‐20:4)	85	80	0.864	0.002	up
	PE(18:0‐20:4)	76	90	0.797	0.002	up
	PE(18:3‐16:0)	94	50	0.794	0.004	up
SM	SM(d18:2/22:1)	88	75	0.865	0.000	up
DG	DG(16:1‐18:3)	76	85	0.851	0.000	up
LPC	LPC(20:4)	76	85	0.838	0.001	up
TG	TG(52:0) NL‐16:0	76	80	0.801	0.025	up

Abbreviations: AUC, area under curve; DG, diacylglycerol; LPC, lysophosphatidylcholine; PC, phosphatidylcholine; PE, phosphatidylethanolamine; SEN, sensitivity; SM, sphingolipid; SPE, specificity; TG, triglycerides.

“:”, carbons and double bonds; “‐”, fatty acyle constituents; “/”, positional isomers.

KEGG pathways analysis between RA and SLE patients was also carried out, and following lipid metabolic pathways were changed such as metabolic pathways, glycerophospholipid metabolism, retrograde endocannabinoid signaling, insulin resistance, glycerolipid metabolism, fat digestion and absorption, and cholesterol metabolism (Figures 2C and 2D).

### 2.4 Characterized lipidomics profile in SLE patients

**FIGURE 3:**
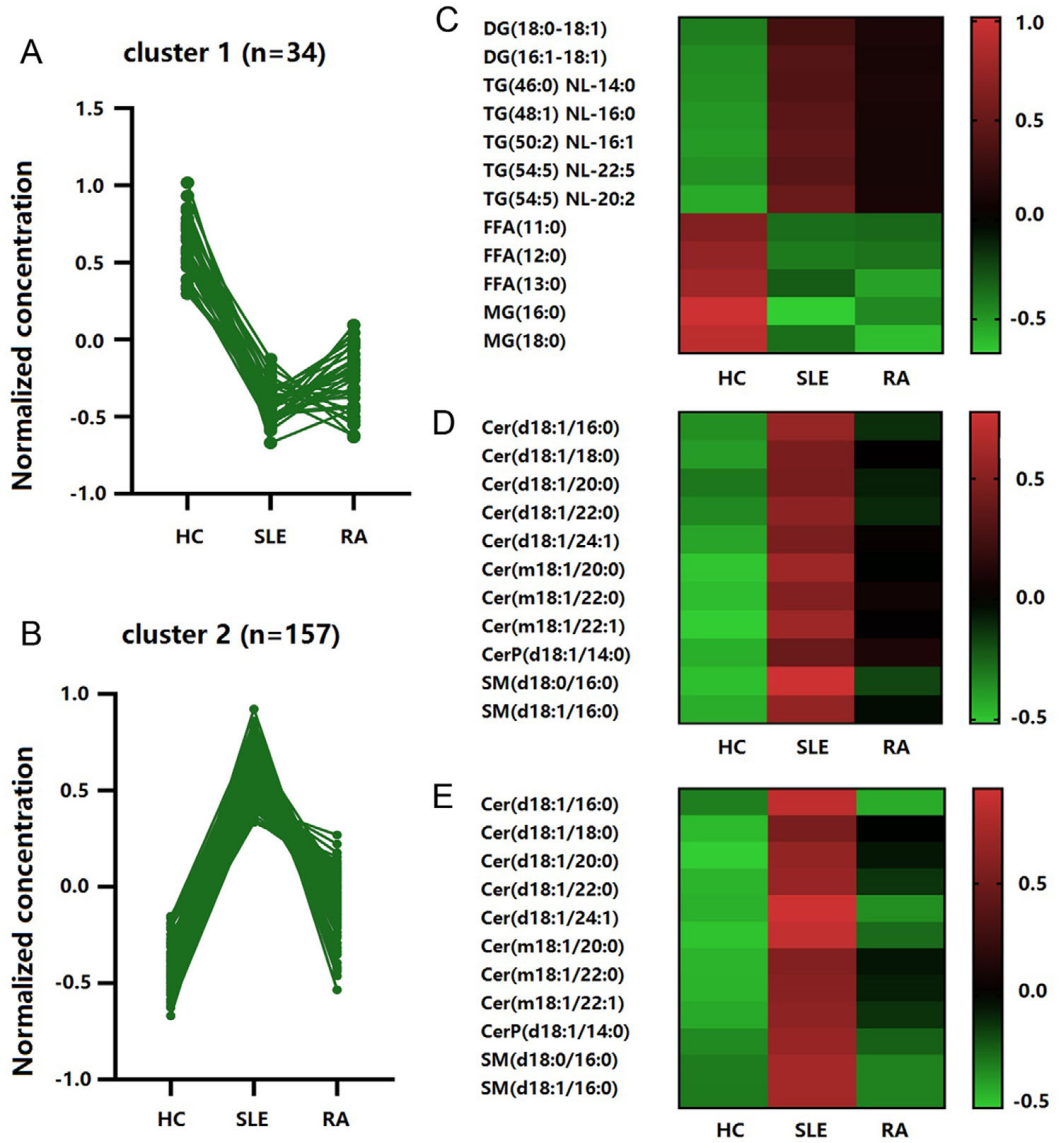
Lipid metabolomic clusters in SLE patients identified by K‐means. (A) Cluster 1 comprised 34 lipid metabolites that showed a significant downward trend in SLE patients. (B) Cluster 2 comprised 157 lipid metabolites that showed a significant upward trend in SLE patients. (C) Triglyceride metabolism lipid metabolites changed in SLE patients. (D) Glycerophospholipid metabolism lipid metabolites were upregulated in SLE patients. (E) Sphingolipid metabolism lipid metabolites were upregulated in SLE patients

The 191 plasma lipid metabolomics which have changed in SLE patients versus HCs were clustered by using k‐means (*n* = 2). In this trial, 34 lipids formed cluster 1, and when compared with HCs, their content in SLE and RA patients has a significant downward trend (Figure 3A); 157 lipids formed cluster 2, and only their content in SLE patients showed a significant upward trend when compared with HCs; however the upward trend in RA patients was unobvious (Figure 3B). In our results, the lipids of cluster 1 and cluster 2 were mainly enriched in the three pathways: triglyceride (TG) metabolism, glycerophospholipid metabolism, and sphingolipid metabolism.

Our results showed that individuals with SLE and RA had significantly lower levels of MG and free fatty acids. when have compared with HCs. We also found significantly higher levels of TGs and diglyceride (DG) only in SLE individuals (Figure 3C). Further analysis showed that both the levels of lysophosphatidylethanolamine, PC, PE in glycerol‐phospholipids metabolism and sphingomyelin (SM), ceramide levels in sphingolipids metabolism were much higher in SLE individuals, while in RA individuals, the increase was much lower, or the level was similar to HCs (Figures 3D) and 3E).

### 2.5 Evaluation of lipid markers on SLE

**FIGURE 4:**
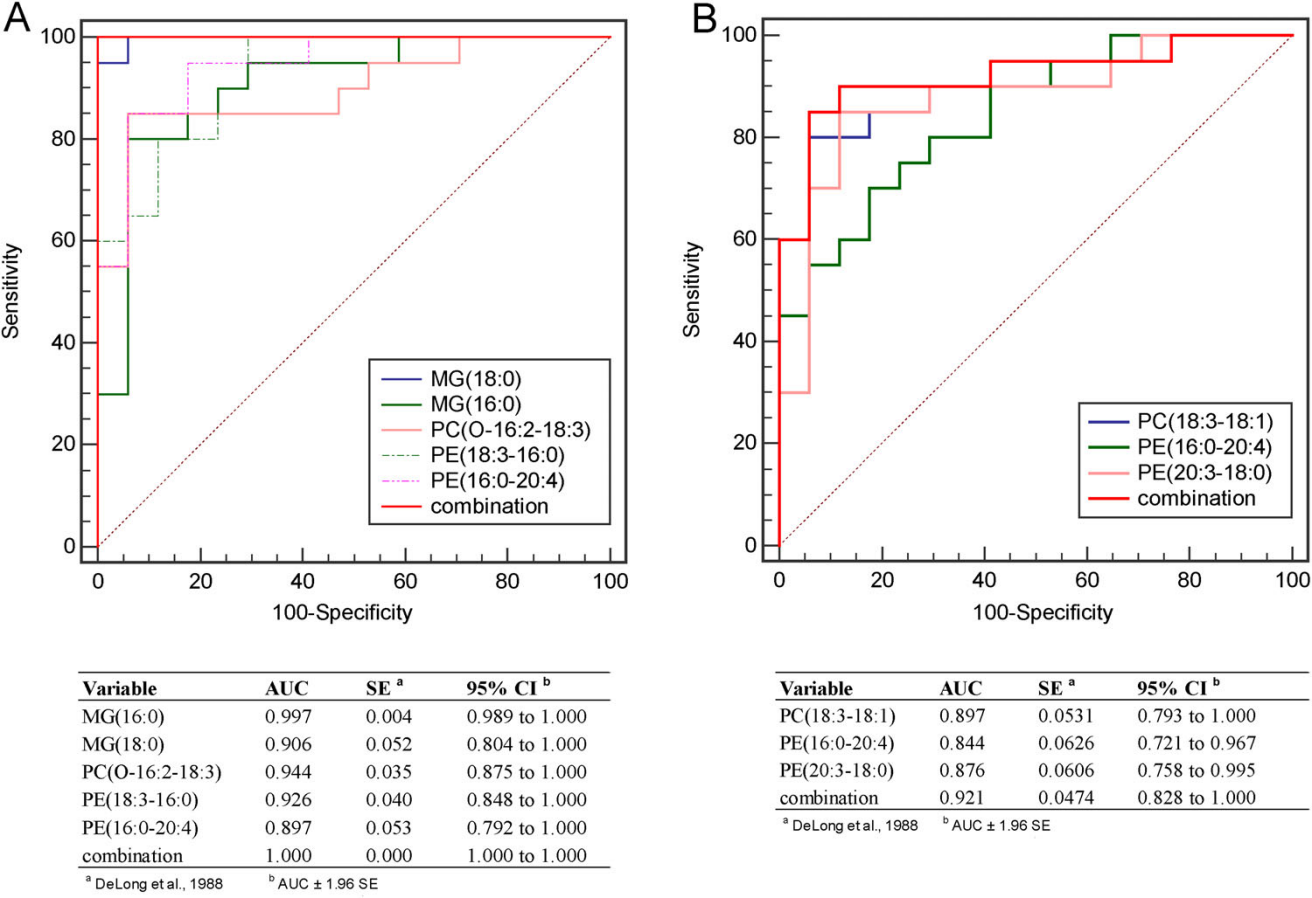
Evaluation potential of lipid metabolites in the diagnosis of SLE from other groups. (A) ROC curve was used to distinguish the patients in the SLE group and HC group; (B) ROC curve was used to distinguish patients in SLE and RA groups

Receiver operating characteristic (ROC) curve was used to evaluate the ability of differential lipids in the diagnosis of SLE patients from RA patients and HCs. The panel of these five differential lipids including MG (16:0), MG (18:0), PE (18:3–16:0), PE (16:0–20:4), and PC (O‐16:2–18:3), has a great potential to distinguish the SLE patients from HC group, with AUC 1.000 (95% CI, 1.000–1.000), specificity 100%, and sensitivity 100% (Figure 4A). Furthermore, the panel of the three differential lipids including PC (18:3‐18:1), PE (20:3‐18:0), and PE (16:0‐20:4) can well distinguish SLE patients from RA patients, with AUC 0.921 (95% CI, 0.828–1.000), 70% specificity, and 100% sensitivity (Figure 4B).

**FIGURE 5:**
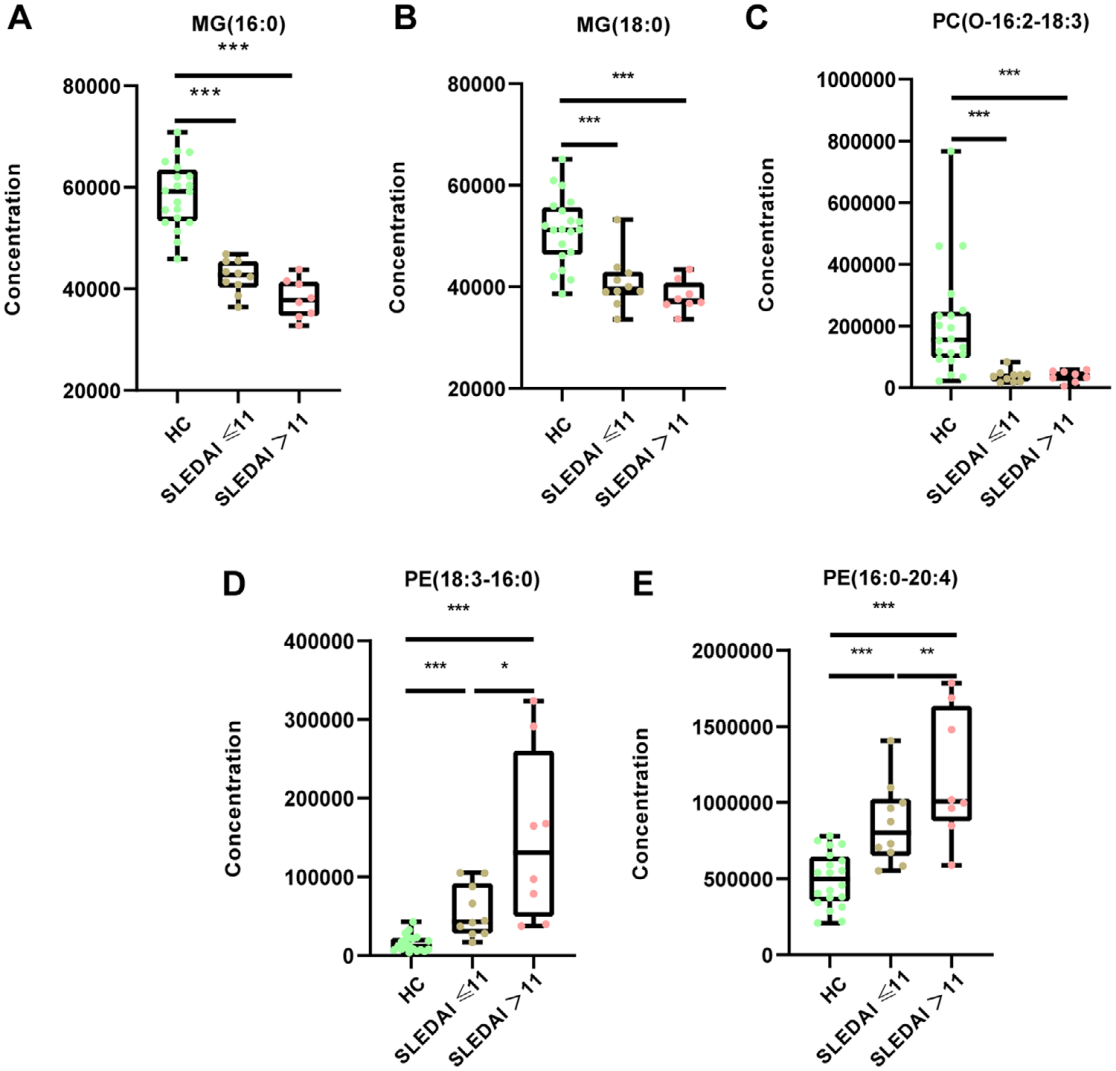
The changes of differential lipids in HC, SLEDAI score ≤ 11 and SLEDAI score > 11 groups, respectively. Relative concentration of (A) MG(16:0), (B) MG(18:0), (C) PC(O‐16:2‐18:3), (D) PE(18:3‐16:0), and (E) PE(16:0‐20:4) in HC, SLEDAI score ≤ 11 and SLEDAI score > 11 groups, respectively. Mann‐Whitney U test was used to test the statistical significance. ***p < 0.001, **p < 0.01, *p < 0.05

Lipid metabolism biomarkers MG (16:0), MG (18:0), and PC (O‐16:2–18:3) were not associated with SLE disease activity index (SLEDAI) score (*p* > 0.05); however, PE (18:3–16:0) and PE (16:0–20:4) were higher in SLEDAI score > 11 group (Figure 5).

## 3 DISCUSSION

The landscape of current SLE candidate biomarkers is relatively narrow.^14^ Therefore, the identification and application of SLE early diagnosis biomarkers are an unmet medical need. Through lipidomic approaches by using UPLC‐MS/MS, it is possible to quantify and identify the level of lipid molecular species, useful to identify biomarkers and to better understand the pathophysiology of SLE. In this study, we described a comprehensive evaluation and revealed a significant lipidomic pattern in SLE patients, with significant changes mainly in phospholipids metabolism, sphingolipids metabolism, and TGs metabolism.

Our results showed dysfunctional sphingolipids metabolism related to the SLE pathophysiology, as the plasma levels of many Ceramide (Cer) and SM increased significantly. Sphingolipids were potential biomarkers for hepatitis B virus (HBV) infection,^15^ Type I diabetes,^16^ and Parkinson's disease.^17^ However, the function of sphingolipids in SLE was unclear. Previous reports illustrated that increased circulating sphingolipids were associated with SLE clinical disease activity.^18^ Recently, Lu et al identified SM with N18:0, N18:1, and N22:0 increased; however, ceramides with N22:0, N23:0, and N24:0 decrease in SLE patients when compared to the HC group.^19^ Studies proved that many sphingolipids elevated in SLE patients’ kidneys and inhibition of sphingolipids synthase could reverse these experimental models.^20^ Besides, sphingolipid‐metabolizing enzymes‐targeted therapies are now used to inhibit autoimmune diseases such as psoriasis and multiple sclerosis.^21^, ^22^ We believe that modulating the sphingolipid pathway might be useful for the future development of therapies given to SLE patients.

Glycerophospholipids contain many derivative forms, which are structural components of cell membranes. Our present results observed upregulated levels of certain glycerophospholipids in SLE patients. The lipids with the most robust differences are shown in PC (18:3–18:1), PE (20:3–18:0), and PE (16:0–20:4), while in RA patients, these levels were similar to healthy subjects, so they permitted to accurately diagnosis of RA from SLE and yielded a classification performance AUC of 0.921. In addition, we found PE (18:3–16:0) and PE (16:0–20:4) were higher in SLEDAI score > 11 group, which might be the important biomarkers for the early diagnosis, prognosis, and treatment of SLE, but further research is needed. Two previous research centers have reported different results, as Hu et al identified PE (16:0–18:1), (16:0–20:4), (18:0–20:4), (18:0–22:4), (18:0–22:5), PC (18:0–18:0), and (16:0–18:2) decreased significantly;^23^ however, Lu et al identified PE species (16:0–18:2), (18:0–18:2), (18:1–18:2), (16:0–22:6), and (18:0–22:6) increased significantly in SLE patients.^19^ Glycerophospholipids are an important part of cell membrane; therefore, these alterations might be associated with changes of lymphocytes membrane and basic defects of immune cell function in immune dysregulation.

Our results gathered herein showed the decrease of some fatty acids and significantly reduction of MG in fat metabolism for SLE patients. Besides, our results also observed the increase of some TG, DG suggests dysfunctional of TGs metabolism. Plasma MG (18:0) and MG (16:0) in both RA and SLE patients decreased significantly. To the best of our knowledge, little information is available on the signaling role of MG and its physiological significance. Monoacylglycerol lipase (MGL) and ABHD6 are the mainly rate‐limiting enzymes that degrade MGs.^24^ MGL hydrolyses TGs or phospholipids into fatty acids and MGs and then regulates lipid accumulation follows inflammation, oxidative stress.^25^, ^26^ Some previous reports have demonstrated the critical role of MGL in cancer development.^27^, ^28^ However, little is known about the role of MGL in SLE. Besides, ABHD6 is also an important contributor to hydrolyses TGs or phospholipids into fatty acids and MGs. Recent GWASs have revealed a robust correlation between ABHD6 and SLE susceptibility.^29^ From our point of view, there might be some possible correlations between the apparent lack of MGs or fatty acids in vivo and enhanced expression of ABHD6 gene in SLE. However, these speculations need to be confirmed by further clinical and biochemical study.

In summary, our large‐scale work makes a step forward in demonstrating the clinical utility of lipidomics signature to assist potentially early diagnosis and treatment SLE. Proposed model of MG (16:0), MG (18:0), PE (18:3–16:0), PE (16:0–20:4) and PC (O‐16:2–18:3) seemed to be ideal for early diagnosis of SLE. Significant changes of lipidomic pattern in phospholipids, sphingolipids and TGs metabolism may reflect some pathological processes or may appear due to ongoing inflammation and damage in the development of SLE. Further validation would be conducted to identify their precise functions on a larger population.

## 4 MATERIAL AND METHODS

### 4.1 Ethical compliance and samples

Ethics approval was obtained from the Ethics Committee of Taizhou Hospital of Zhejiang Province. All the investigation protocols were carried out in accordance with the relevant guidelines and complied with the principles of the Declaration of Helsinki. Written informed consent was achieved from each participant for their blood to be used for this research.

Between May 2019 and October 2019, 18 SLE patients, 20 RA patients, and 20 healthy subjects matched for gender, age, and ethnicity were recruited from Taizhou Hospital of Zhejiang Province. The diagnosis for RA and SLE patients was strictly according to the criteria,^30^, ^31^ and their clinical information on gender, age, BMI, and laboratory inflammation indicators was available. We used rigorous inclusion criteria for HCs with no cardiovascular, tumors, immunity, or other known infection diseases.

Approximately 2 ml EDTA anticoagulated blood was collected from every subject under fasting conditions. The plasma samples were immediately stored at ‐80°C after centrifugation for further UPLC‐MS/MS processing and analysis.

### 4.2 Lipidomics analyses

Firstly, the plasma samples were thawed at room temperature, vortexed for 10 s, and centrifuged at 3000 rpm for 5 min at 4°C. Secondly, transfer 50 ul of sample per tube to a new EP tube, mix with 1 mL of lipid extraction solution, and vortex for 2 min. Thirdly, the ultrasound tube was sonicated for 5 min, mixed with 500 uL of water, vortexed for 1 min, and centrifuged at 12000 g at 4°C for 10 min. Then collect 500 ul of supernatant and dry with nitrogen, reconstitute with 100 ul of mobile phase B. The sample was vortexed for 1 min and then centrifuged at 14000 g at 4°C for 15 min. Finally, the samples were analyzed by UPLC‐MS/MS.

The calibration and quality control (QC) samples were prepared with the mixed plasma of subjects prior to sample analysis. Every 10 samples to be analyzed were separated by one QC sample for the duration of the detection to monitor repeatability during the analysis. The high overlaps of the total ion flow between different QC samples, that is, the retention time and peak strength are consistent, indicate that the signal stability of the mass spectrum is good at different times.

### 4.3 UPLC‐MS/MS analysis

The lipid metabolites of plasma samples were initially separated on an ultra‐high performance liquid chromatograph (Thermo C30, i.d.2.1 × 100 mm 2.6 um columns). The column temperature was set to 45°C. The mobile phase (A) uses 60% acetonitrile in water, and the mobile phase (B) uses 10% acetonitrile in isopropanol. The elution gradient at 0 min is set to 20% B, the 3 min elution gradient is set to 50% B, the 9 min elution gradient is set to 75% B, and the 15 min elution gradient is set to 90% B liquid, the balance liquid is 50% B liquid, the flow rate is set to 350 ul/min, and the injection volume of sample was 2 μl.

Then initially separated sample enters the QTRAP LC‐MS / MS system. The system is equipped with an ESI Turbo ion spray interface and can be operated in positive and negative ion modes. The operating parameters of the ESI source are as follows: the ion source temperature is set to 550°C; the ion spray voltage is set to 5500 V; the ion source gas I, gas II, and curtain gas are set to 55, 60, and 25 psi, and the collision gas is set to medium. Note that 10 and 100 μmol/L polypropylene glycol solutions were used for instrument tuning and PPG calibration separately. Quantitative analysis is accomplished by multiple reaction monitoring analysis. The signal intensity of characteristic ions is obtained and represents the relative content of corresponding substance. MultiQuant is used to integrate and correct chromatographic peaks.

### 4.4 Statistical analysis

The software Analyst 1.6.3 was used to process the mass spectrometry data. The classification and naming of lipids are strictly in accordance with LIPID MAPS convention (www.lipidmaps.org). The baseline characteristics of the study population were evaluated by chi‐square test and Kruskal‐Wallis H test. MetaboAnalyst 4.0 (http://www.metaboanalyst.ca) was applied to analyze the metabolite OPLS model. R2X (the interpretability of the model for the categorical variable X), R2Y (the interpretability of the model for variable Y), and Q2 (the predictability of the model) were obtained to judge the validity of OPLS model. K‐means clustering method was used to screen the lipid metabolites of SLE group, RA group, and the HC group. The binary logistic regression fitting to evaluate the diagnostic value of the combined model and the MedCalc (19.0.7) software were used to draw the receiver characteristic working curve (ROC) which includes AUC value, sensitivity, and specificity.

## AUTHOR CONTRIBUTIONS

Jiaxi Chen conceived the idea and designed the experiments. Shenyi Ye, Xianhong Ding, and Yang Lu carried out experiments. Haiyan Lv, Xiangyu Xu, Bingjie Hu, and Chenliang Hong analyzed experimental results. Jiaxi Chen, Chong Liu, and Jiaqin Xu wrote the main manuscript. Ruyue Lu, Hongguo Zhu, and Bo Shen supervised all aspects of the study. All authors reviewed the manuscript.

## CONFLICT OF INTEREST

All authors declare no competing interests.

## ETHICS STATEMENT

This project was permitted by the Ethics Committee of Taizhou Hospital of Zhejiang Province. This research conforms to all the laws and ethical guidelines that apply in the country.

## Data Availability

The datasets used and analyzed during the current study are available from the corresponding author upon reasonable request.
